# Enhanced probabilistic prediction of pavement deterioration using Bayesian neural networks and cuckoo search optimization

**DOI:** 10.1038/s41598-025-92469-9

**Published:** 2025-03-13

**Authors:** Feng Xiao, Biying Shi, Jie Gao, Huapeng Chen, Di Yang

**Affiliations:** 1 School of Transportation Engineering, East China JiaoTong University, Nanchang, 330013 China; 2 School of Architectural and Art, Jiangxi Industry Polytechnic College, Nanchang, 330000 China; 3 School of Civil Engineering and Architecture, East China JiaoTong University, Nanchang, 330013 China; 4 Institute of Transportation Development Strategy & Planning of Sichuan Province, Chengdu, 610041 China

**Keywords:** Pavement management systems, Deterioration, Probabilistic prediction, Bayesian neural network, Cuckoo search algorithm, Civil engineering, Information technology

## Abstract

The predictive performance of probabilistic pavement condition deterioration is critical for effective maintenance and rehabilitation decisions. Currently, numerous improved models exist, but few rely on probabilistic models to improve pavement deterioration prediction. Therefore, this study proposed an improved probabilistic model for pavement deterioration prediction based on the coupling of Bayesian neural network (BNN) and cuckoo search (CS) algorithm. The model prediction performance is evaluated against two metrics: determination coefficient (R^2^) and standard deviation (stability). Finally, based on the data from the pavement management system in Shanxi Province, it was verified that the CS-BNN model outperforms the genetic algorithm-BNN, particle swarm optimization-BNN, and BNN models in terms of the two metrics. Sensitivity analysis further confirms the robustness of the CS-BNN model. The findings indicate that the CS-BNN model provides more reliable predictions with lower uncertainty, aiding road engineers in optimizing maintenance schedules and costs.

## Introduction

Pavement condition prediction plays a crucial role in the field of transportation infrastructure management. Accurate prediction of road pavement condition deterioration significantly impacts the development of annual maintenance and rehabilitation decision-making plans^1–4^. A reliable pavement condition deterioration prediction model is essential not only for optimizing maintenance and rehabilitation costs but also for ensuring the safety and serviceability of road networks for users.

Despite the availability of various pavement condition deterioration prediction models, there is a critical need for improved probabilistic models that address predictive accuracy and uncertainty robustness. Existing deterministic models fail to account for the inherent uncertainty in pavement evolution, while tradition probabilistic approaches often produce predictions with poor stability under varying conditions. These limitations can lead to suboptimal maintenance and rehabilitation decisions. Deterministic models tend to maintenance and rehabilitation decisions that underestimate the required costs and overestimate the post-maintenance pavement condition levels^5^, whereas current probabilistic prediction models often result in maintenance and rehabilitation decisions that overestimate the required costs and underestimate the post-maintenance pavement condition levels.

To address this gap, this study proposed a novel Cuckoo Search-Bayesian Neural Network (CS-BNN) model, which combines the global optimization capabilities of the CS algorithm with the probabilistic framework of BNNs. The primary objectives of this study are to develop a probabilistic pavement condition deterioration prediction model that improves predictive accuracy and uncertainty robustness. The proposed prediction model is a more reliable and accurate tool for pavement infrastructure management, ultimately contributing to more cost-effective and timely maintenance decisions.

## Literature review

Over the years, numerous methods have been developed for pavement condition prediction. These can be broadly classified into several categories. For example, Uddin^6^ categorized them as deterministic, probabilistic and artificial neural network models. The Pavement Management Guide^7^ grouped them into deterministic, probabilistic, Bayesian, and subjective (or expert-based) models. Pavement performance prediction models can also be classified into deterministic, probabilistic, and hybrid types^8^. Among these models, the deterministic and probabilistic models attract the greatest attention^9,10^.

Deterministic models are models where a predicted variable (or variables) are obtained from some independent variables, generally by mean of regression analysis^7^. The advantage of deterministic models lies in their simplicity and interpretability. However, they fail to account for the inherent uncertainty in pavement evolution, as pavement behavior is recognized to be probabilistic in nature^11–14^.

To address this uncertainty, probabilistic model have been developed. The stochastic process approach, which includes Markov methods and Gaussian process regression^15,16^, is commonly used. Markov-based models rely on the transition probability matrix to predict the transfer of pavement states. The transition probability matrix can be obtained through various methods such as back-calculation^13^, empirical methods^17^, optimization algorithms^18^ and simulation-based approaches^19,20^. Although they have an advantage in computational complexity, they are limited to a finite number of discrete variables, while most pavement condition indicators are continuous. Gaussian process regression, on the other hand, is a non-parametric model that can handle continuous values. It has been used in various aspects of pavement prediction, such as predicting the international roughness index (IRI) of flexible pavements^21^ and estimating the structural capacity of flexible pavements^22^. Gaussian process regression excels at modeling complex, non-linear relationships and provides uncertainty estimates, but it suffers from high computational complexity, scales poorly with large datasets, and is highly sensitive to the choice of kernel and hyperparameters.

In recent decades, machine learning algorithms have gained significant attention in pavement performance prediction^8,23–25^. Neural network models, a typical type in machine learning, have shown high prediction accuracy. Guo et al.^26^ constructed a multi-output prediction model for rigid pavement deterioration using the correlation between four pavement condition indicators (IRI, faulting, longitudinal crack and transverse crack) and the neural network theory, and verified that the prediction accuracy of the multi-output model is higher than that of the single-output model. Recurrent neural networks, another type of neural network-related method, are suitable for road pavement data with time series characteristics. Sun et al.^27^ established the semi-rigid asphalt pavement performance multi-output prediction model based on Long Short-Term Memory. In addition, the transformer model, which is also a type of neural network-related method, has better prediction accuracy. Han et al.^28^ proposed a predictive model for road health indicators (rutting depth, surface texture depth, center point deflection and deflection basin area of 5t falling weight deflectometer) based on improved transformer network, and they validated that the proposed model had higher predictive accuracy than recurrent neural network-based and artificial neural network-based prediction models. Although these neural network-related methods are able to achieve high prediction accuracy, the mathematical mapping from inputs to output in neural networks is not easily interpreted by humans, leading to low trustworthiness of its applications in practices. Moreover, these neural network-related methods, like deterministic models, are unable to address the uncertainty in the pavement evolution process.

By combining neural networks with Bayesian theory, a probabilistic Bayesian neural network (BNN) can be further obtained. The BNN can be used to develop probabilistic pavement performance prediction models, which are modeled in two way: probabilistic weights and structure^29^. For the BNN with probabilistic weights, its weight values are not definite values but probability distributions^30^. Thus, the same set of input values may correspond to different output values. For the BNN with probabilistic structure, the dropout is applied at both training and testing processes, resulting in a variable (i.e., probabilistic) model structure^31^. BNNs offer advantages such as the ability to quantify uncertainty, which is crucial in pavement performance prediction.

Furthermore, some researchers had tried to improve the pavement performance prediction models by relying on existing algorithms^32–34^. Wang and Li^35^ proposed a fuzzy regression method to determine the coefficients of the gray prediction model, so as to construct a fuzzy and gray-based IRI prediction model. They proved that the hybrid model was superior to the Mechanistic-Empirical Pavement Design Guide model and gray models. Deng and Shi^36^ coupled feed-forward neural networks with particle swarm optimization to obtain an improved pavement rutting prediction model. They verified that the improved model achieved better performance in terms of accuracy, reproducibility, and robustness. Similarly, there are other improved pavement performance prediction models, such as, the neural network model optimized by genetic algorithms^37–39^, the support vector regression model optimized by particle filter^40^ and by genetic algorithm^41^, and the gene expression programming-neural network model^42^.

Despite these advancements, there is a significant gap in the development of probabilistic prediction models that focus on prediction accuracy and uncertainty robustness simultaneously. Therefore, this study proposed a novel probabilistic pavement condition deterioration prediction model.

Traditional probabilistic models, such as Markov-based models and Gaussian process regression, are either limited in their ability to handle continuous variables or suffer from high computational complexity. In contrast, BNNs combine the expressive power of neural networks with the probabilistic framework of Bayesian methods, enabling them to capture complex nonlinear relationships and quantify uncertainty effectively^24,43^. These properties make BNNs particularly well-suited for addressing the uncertainty associated with pavement condition evolution, motivating our choice to improve upon this model. To further improve the probabilistic model, the authors attempted to combine an optimization algorithm with a BNN to form a new hybrid prediction model.

The Cuckoo search (CS) algorithm is a nature-inspired optimization algorithm^44^. It has several advantages compared with other optimization methods like the Genetic Algorithm (GA) and the Particle Swarm Optimization (PSO) algorithm. The CS algorithm has a better balance between exploration and exploitation, which means it can search a wider solution space in the initial stages and then focus on refining the best-found solution more effectively. It also has fewer parameters to adjust, making it more convenient to use. In addition, it had shown better performance in dealing with complex optimization problems^45,46^. Therefore, the CS was chosen to be combined with the BNN to improve probabilistic pavement condition deterioration prediction models in terms of the goodness-of-fit and stability.

## Data collection and methods

### Data collection

In this study, data related to asphalt pavements of freeways in the Shanxi Pavement Management System were used to validate pavement condition deterioration prediction models. The data variables are described in Table 1. In this study, a total of 5223 roadway pavement data were collected, of which randomly 80% were used as training data and 20% as validation data.

**Table 1:** The data variables.

Variables	Description	Variable type
Structure and material data		
Thickness of surface layers	Sum of thickness of upper, middle, and lower surface layers in Fig. 1	Numerical
Materials of base courses	Mixtures of cement, lime, industrial waste, or asphalt with soil or gravel	Categorical
Traffic volume data		
AADTT	Average annual daily truck traffic	Numerical
AADT	Average annual daily traffic	Numerical
Climate and environmental data		
Total high temperature days	Total number of days with daily average temperature > 25 ℃ in a year	Numerical
Number of consecutive high temperature	Number of consecutive 3 days or more with daily average temperature > 25 ℃ in a year	Numerical
Total low temperature days	Total number of days with daily average temperature < 0 ℃ in a year	Numerical
Number of consecutive low temperature	Number of consecutive 3 days or more with daily average temperature < 0 ℃ in a year	Numerical
Pavement condition data		
PCI of previous year	PCI: Pavement surface condition index, with values ranging from 0 to 100, where 0 indicates the worst pavement condition and 100 indicates the best pavement condition	Numerical
PCI of current year		Numerical
Other data		
Road age	Current year—year of road completion	Numerical

**Fig. 1:**
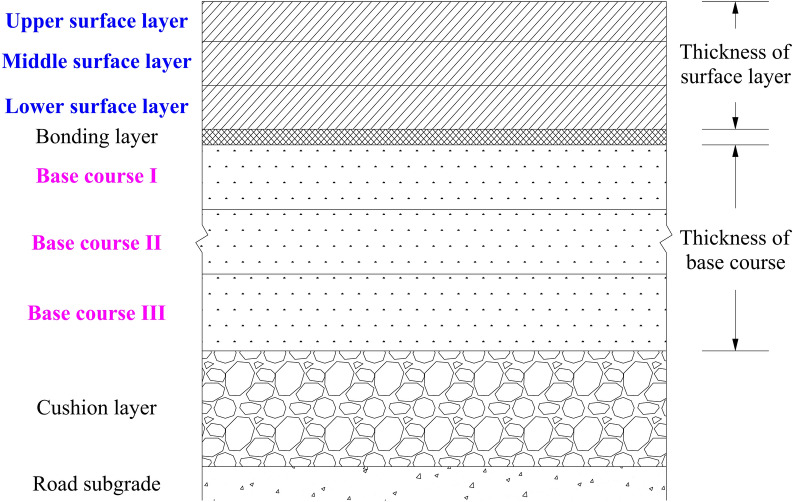
A freeway cross-section in Shanxi Province.

For the base courses, although three courses are shown in Fig. 1, two courses (Base courses I and II) are also a common structural type. In the collected data, the materials of base courses are usually mixtures of cement, lime, industrial waste or asphalt with soil or gravel. So, it has five different combinations: mixtures of cement with soil or gravel (base course I and II), mixtures of cement with soil or gravel (base course I) + mixtures of lime with soil or gravel (base course II), mixtures of cement with soil or gravel (base course I) + mixtures of industrial waste with soil or gravel (base course II), mixtures of asphalt with soil or gravel (base course I) + mixtures of cement with soil or gravel (base courses II and III), and mixtures of lime with soil or gravel (base course I) + mixtures of cement with soil or gravel (base course II) + mixtures of industrial waste with soil or gravel (base course III).

According to China’s highway climate zoning standards, Shanxi Province is located in the dry and wet transition zone of the Loess Plateau. The daily average temperature > 25 ℃ and < 0 ℃ are defined as high and low temperatures, respectively. In the definition of the number of consecutive high/low temperatures, the 3-day threshold is based on the experience of local engineers.

To help readers further understand the above variables, Table 2 shows descriptive statistics for numerical variables.

**Table 2:** Descriptive statistics of numerical variables.

Name	Unit	Mean	Standard deviation	Minimum	Maximum	Median
Thickness of surface layers	m	0.18	0.03	0.1	0.22	0.17
AADTT	Vehicles	3611.51	2863.19	301	13,564	3175
AADT	Vehicles	8870.38	6402.53	871	31,485	7110
Total high temperature days	Days	69.95	34.08	3	106	86
Number of consecutive high temperature	–	10.08	4.06	1	16	12
Total low temperature days	Days	131.23	38.06	73	177	128
Number of consecutive low temperature	–	7.39	3.22	2	12	7
PCI of previous year	–	90.05	6.85	57.6	100	91.4
Road age	Years	10.89	6.19	1	25	9
PCI of current year	–	84.81	8.67	46.6	99.35	86.3

### Proposed prediction model

The core of the proposed prediction model is the coupling of a Bayesian neural network with a cuckoo search algorithm, so it is necessary to introduce the Bayesian neural network and cuckoo search algorithm.

#### Bayesian neural network

For a given training set $D=\left\{X,Y\right\}$, the key to obtain the probability distribution of weights $P\left(w|D\right)$. Under the Bayesian framework, $P\left(w|D\right)$ is expressed by Eq. (1).

1 $$P\left(w|D\right)=\frac{P\left(D|w\right)P\left(w\right)}{P\left(D\right)}$$

where $P\left(w\right)$ is a prior distribution; $P\left(D|w\right)$ is a model likelihood; $P\left(D\right)$ is a marginal likelihood; and $w$ is model weights. Prior distribution represents initial belief or assumption about the possible values of a model parameter before observing any data. Likelihood measures the probability of observing the current data given specific parameter values, reflecting how well the parameters explain the data.

For a new input ${x}^{*}$ and an output ${y}^{*}$, the predictive distribution is defined as Eq. (2).

2 $$P\left({y}^{*}|{x}^{*},D\right)=\int P\left({y}^{*}|{x}^{*},w\right)P\left(w|D\right)dw$$

However, $P\left(w|D\right)$ is intractable for any real-scale neural networks^47^. Instead, the posterior distribution of $P\left(w|D\right)$ can be approximated by a simple distribution of $q\left(w|\theta \right)$, parameterized by $\theta$. Naturally, $P\left(w|D\right)$ and $q\left(w|\theta \right)$ are expected to as similar to each other as possible, and the difference between the two distributions is measured using the Kullback–Leibler (KL) divergence, i.e., minimizing the KL divergence value, as shown in Eq. (3).

3 $${\theta }^{*}=\text{arg}\underset{\theta }{\text{min}}KL\left[q\left(w|\theta \right)||P\left(w|D\right)\right]$$

Further derivation of Eq. (3) yields Eqs. (4)–(6).

4 $${\theta }^{*}=\text{arg}\underset{\theta }{\text{min}}{\mathbb{E}}_{q\left(w|\theta \right)}\left\{\text{log}\left[\frac{q\left(w|\theta \right)}{P\left(w|D\right)}\right]\right\}$$

5 $${\theta }^{*}=\text{arg}\underset{\theta }{\text{min}}{\mathbb{E}}_{q\left(w|\theta \right)}\left\{\text{log}\left[\frac{q\left(w|\theta \right)P\left(D\right)}{P\left(D|w\right)P\left(w\right)}\right]\right\}$$

6 $${\theta }^{*}=\text{arg}\underset{\theta }{\text{min}}{\mathbb{E}}_{q\left(w|\theta \right)}\left\{\text{log}\left[\frac{q\left(w|\theta \right)}{P\left(D|w\right)P\left(w\right)}\right]\right\}$$

Then, the minimization objective is $\mathcal{F}\left(D,\theta \right)$, as shown in Eq. (7).

7 $$\mathcal{F}\left(D,\theta \right)={\mathbb{E}}_{q\left(w|\theta \right)}\left\{\text{log}\left[\frac{q\left(w|\theta \right)}{P\left(D|w\right)P\left(w\right)}\right]\right\}$$

**Fig. 2:**
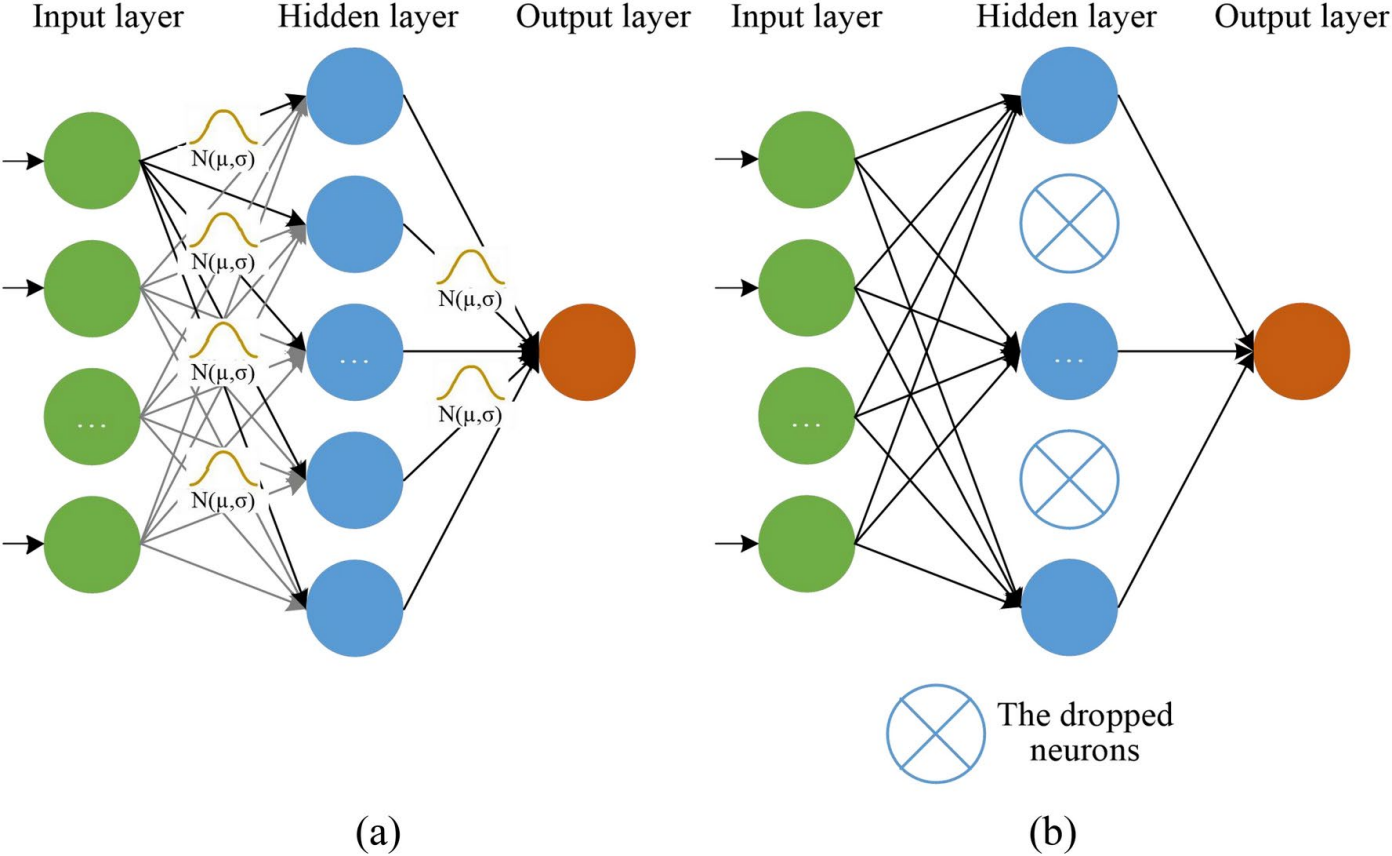
Schematic of two BNNs with (**a**) probabilistic weights and (**b**) probabilistic structure.

The variational distribution $q\left(w|\theta \right)$ is usually recommended to use the product of Gaussian distributions^47^. Then, the BNN with with probabilistic weights can be obtained^30^, as shown in Fig. 2a. However, the approach performs poorly in practice due to the large number of weight parameters required. In contrast, Monte Carlo dropout is another simpler method of variational inference by a product of Bernoulli distribution. Monte Carlo dropout applies dropout at both training and testing steps, i.e., the BNN with probabilistic structure in Fig. 2b.

#### Cuckoo search algorithm

**Fig. 3:**
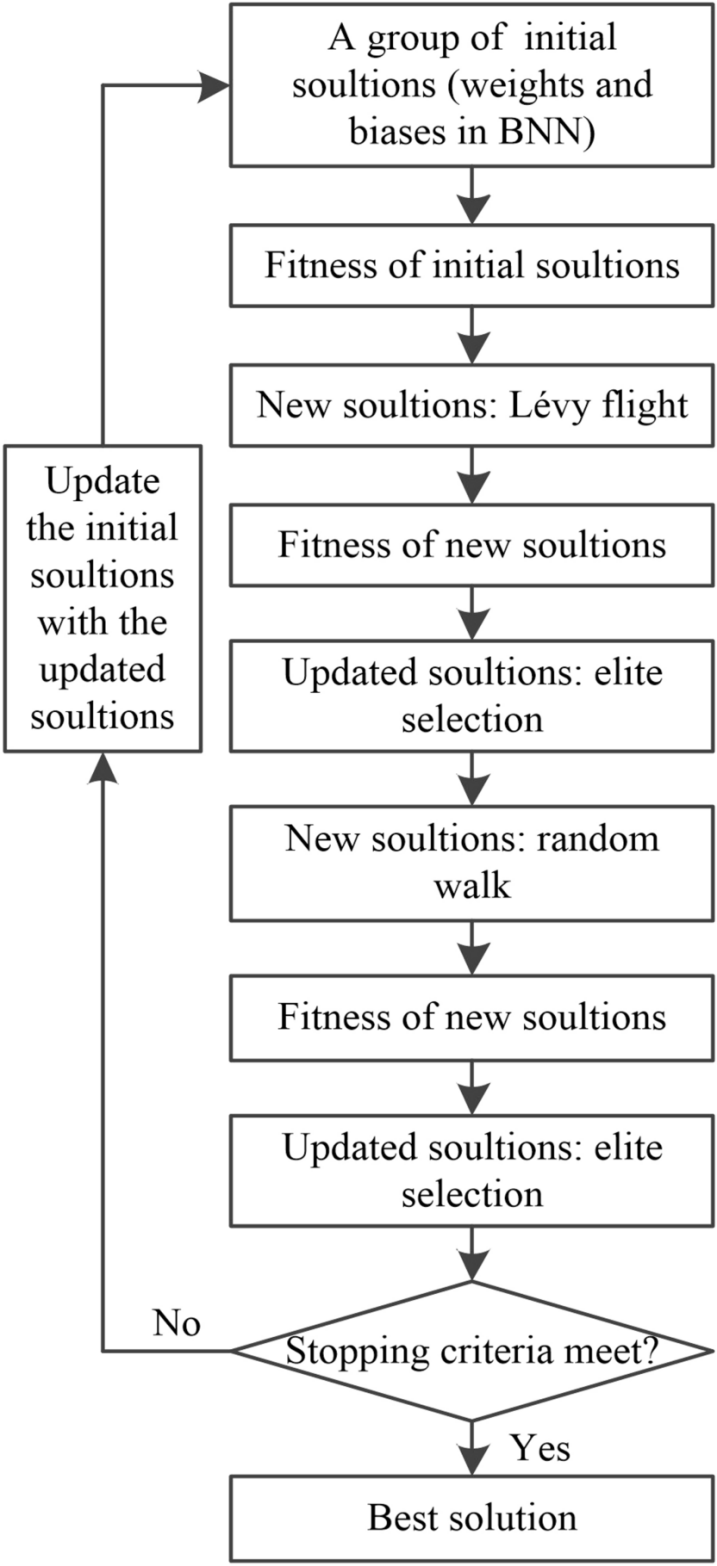
Processes of cuckoo search algorithm.

The CS algorithm in this study consists of five parts in Fig. 3^44–46^:

1. Generation of a group of initial solutions. Each element in a solution represents a weight or bias value in BNN, as shown in Fig. 4. The initial solutions are randomly sampled on a truncated normal distribution.

**Fig. 4:**
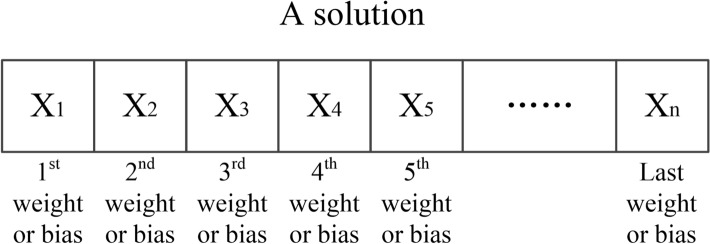
A schematic diagram of a solution.
2. Computation of fitness. The weight and bias values in each solution are substituted into the BNN model for training, and the determination coefficient of the BNN model is the fitness value.
3. Creation of new solutions by Lévy flight. When generating new solutions, the theoretical formulae of Lévy flight are shown in Eqs. (8)–(12).
  8 $$x_{i}^{{\left( {g + 1} \right)}} = x_{i}^{{\left( g \right)}} + \alpha \otimes {\text{L}}\mathop {\text{e}}\limits^{\prime } {\text{vy}}\left( \beta \right)$$
  in which:
  9 $${\text{L}}\mathop {\text{e}}\limits^{\prime } {\text{vy}}\left( \beta \right) = \frac{\mu }{{\left| v \right|^{{\left( {1/\beta } \right)}} }}$$
  10 $$\mu \sim N\left(0,{\sigma }^{2}\right)$$
  11 $$v\sim N\left(\text{0,1}\right)$$
  12 $$\sigma ={\left\{\frac{\Gamma \left(1+\beta \right)\text{sin}\left(\frac{\pi \beta }{2}\right)}{\beta\Gamma \left(\frac{1+\beta }{2}\right){2}^{\frac{\beta -1}{2}}}\right\}}^{\frac{1}{\beta }}$$
  where ${x}_{i}^{\left(g\right)}$ is the ${i}^{th}$ element of the ${g}^{th}$ generation in a solution; ${x}_{i}^{\left(g+1\right)}$ is the ${i}^{th}$ element of the ${\left(g+1\right)}^{th}$ generation in a solution; $\alpha >0$ is the step size; generally, $\alpha =1$; the product $\otimes$ is entry-wise multiplications; $\mu$, $v$ are random values subject to normal distributions; $\Gamma \left(z\right)$ is the Gamma function, and $\Gamma \left(z\right)={\int }_{0}^{+\infty }{t}^{z-1}{e}^{-t}dt$. $\beta$ is a constant value, and $\beta =1.5$.
4. Creation of new solutions by random walk. When generating new solutions, the theoretical formula of random walk is shown in Eq. (13).
  13 $${x}_{i}^{\left(g+1\right)}={x}_{i}^{\left(g\right)}+\alpha \otimes \text{H}\left({P}_{a}-\varepsilon \right)\otimes \left[{x}_{j}^{\left(g\right)}-{x}_{k}^{\left(g\right)}\right]$$
  where ${x}_{j}^{\left(g\right)}$ and ${x}_{k}^{\left(g\right)}$ are two different solutions selected by random permutation at the ${g}^{th}$ generation; ${P}_{a}$ denotes the probability of being updated by the random walk. $\text{H}\left(z\right)$ is a Heaviside function; $\varepsilon$ is a random value sampled from a standard normal distribution.
5. Update of solutions by elite selection. After the operations of Lévy flight and random walk, new solutions are obtained. The two fitness values of each new solution and each old solution are compared in sequence. If the fitness value of the new solution is better than the old solution, the new solution replaces the old solution; otherwise, the old solution remains unchanged.

#### Pavement deterioration prediction model based on CS-BNN

**Fig. 5:**
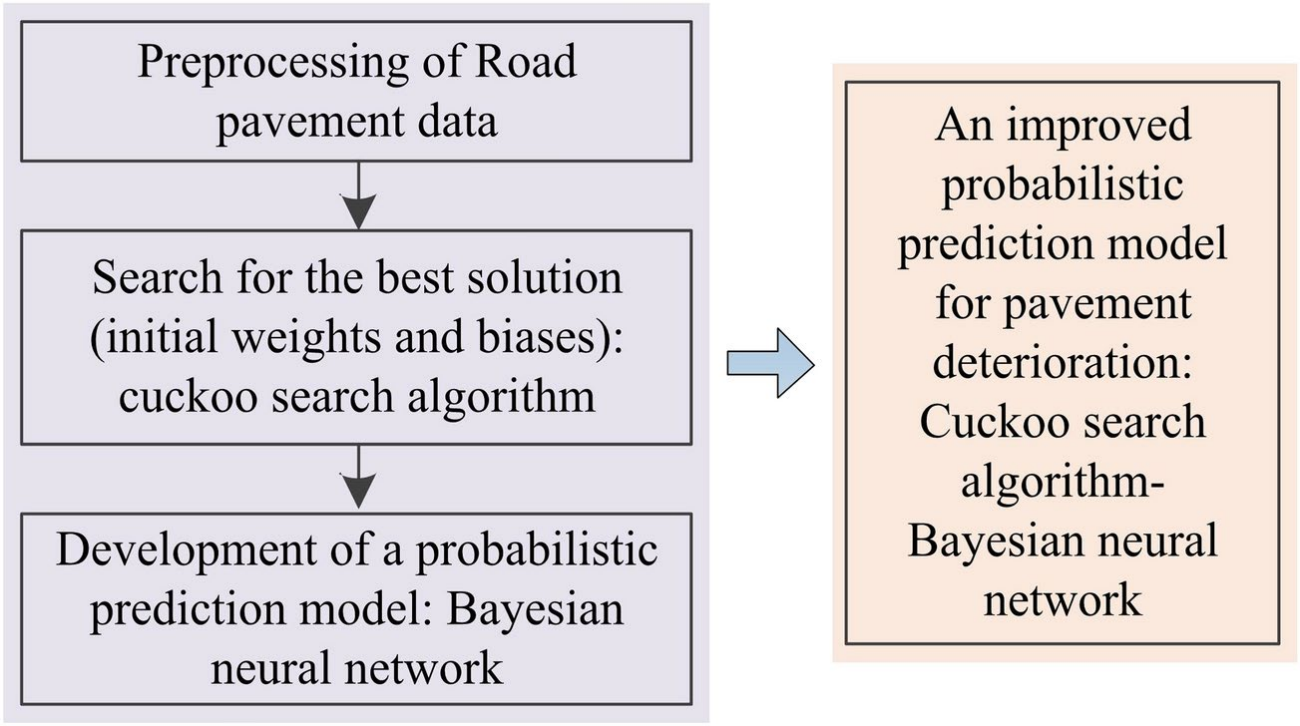
Processes of the proposed CS-BNN prediction model.

The development of the proposed prediction model consists of three steps (Fig. 5). Firstly, the road pavement data are preprocessed in two steps: data cleaning and normalization. Road pavement data containing outliers (e.g., cases where the road surface is not be treated but the condition unexpectedly improves) or missing values are removed. The 5223 road pavement data mentioned before are the cleaned data. And the road pavement data are normalized using z-score method. The theoretical equation for the z-score method is shown in Eq. (14).

14 $${x}_{k}^{*}=\frac{{x}_{k}-{\mu }_{k}}{{\sigma }_{k}}$$

where ${x}_{k}^{*}$, ${x}_{k}$, ${\mu }_{k}$, ${\sigma }_{k}$ are normalized value, initial value, mean, standard deviation of the ${k}^{\text{th}}$ variable, respectively.

Then, the best initial solution (i.e., weights and biases for BNN) is searched by the CS algorithm. The specific steps are (a) randomly generating multiple sets of solutions (weights and biases), (b) maximizing the determination coefficient of the BNN during the training process as the optimization objective, and (c) searching for a set of optimal initial weight and bias values by CS.

Finally, the pavement deterioration prediction model is constructed based on BNN. Using the best initial solution and preprocessed data involving road pavement, the BNN model (which incorporates Monte Carlo dropout during both training and testing) is retrained to finally obtain an improved probabilistic prediction model for pavement deterioration.

### Comparison of the proposed prediction model

To verify the superiority of the proposed prediction model, three comparison models were developed^48^. Constructing a BNN-based pavement deterioration prediction model as the first comparison model can visualize the advantages brought by the CS algorithm. Since existing studies indicate that GA^37^ and PSO^36^ have similar advantages to the CS algorithm, pavement deterioration prediction models based on GA-BNN and PSO-BNN are constructed as the second and third comparative models. The detailed implementation of the BNN, GA-BNN, and PSO-BNN is provided in ESM Appendix A to ensure transparency and fairness in the comparison.

#### Pavement deterioration prediction model based on BNN

In contrast to the proposed prediction model, the initial weights and biases of the BNN-based model are sampled from a truncated normal distribution instead of being obtained by the CS algorithm. The rest of the modeling steps are the same as the proposed prediction model.

#### Pavement deterioration prediction model based on GA-BNN

**Fig. 6:**
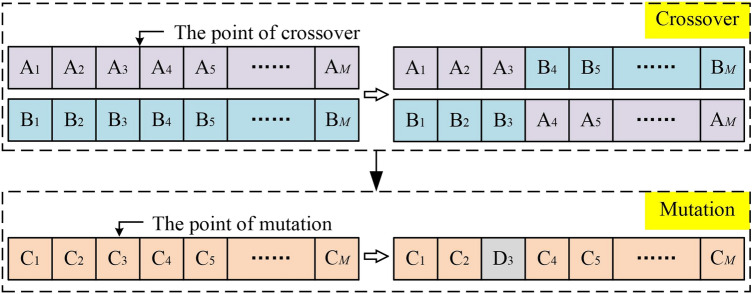
The crossover and mutation operations.

In contrast to the proposed prediction model, the GA-BNN model generates next-generation solutions through crossover and mutation operations, rather than Lévy flight and random walk. The rest of the modeling steps are the same as the proposed prediction model. The crossover and mutation operations in a GA are schematically shown in Fig. 6.

#### Pavement deterioration prediction model based on PSO-BNN

In the process of searching for the best solution, the PSO-BNN model generates the next generation of solutions by utilizing individual extremes and population extremes^49^, rather than Lévy flight and random walk. The rest of the modeling steps are the same as the proposed prediction model.

**Fig. 7:**
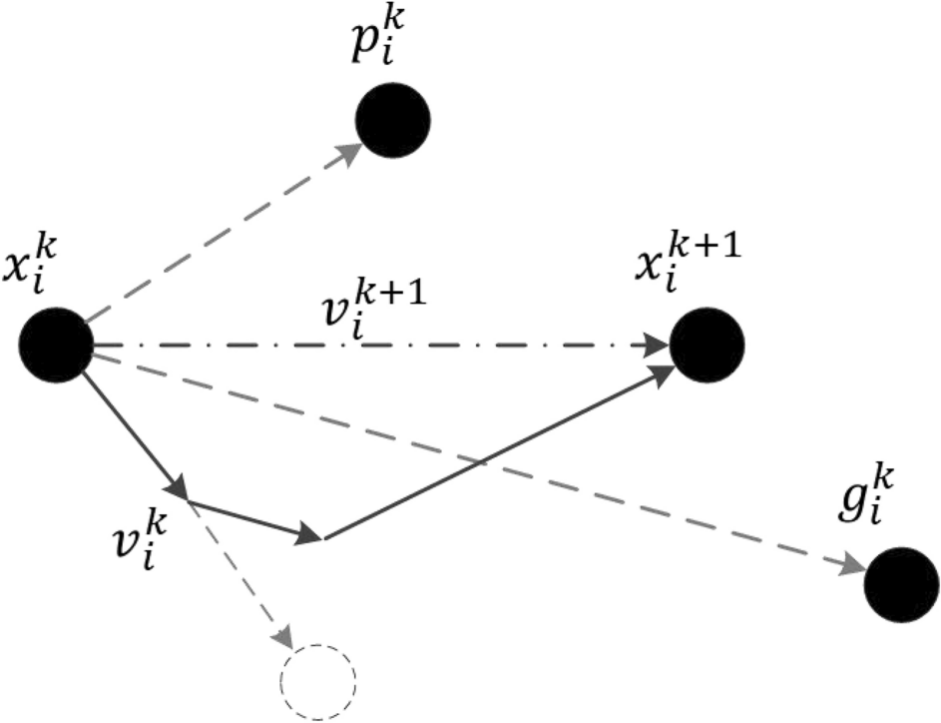
Processes of generating next-generation solutions in PSO.

The theoretical expressions for generating next generation solutions are shown in Eqs. (15) and (16), and the processes are graphically illustrated in Fig. 7.

15 $${x}_{i}^{k+1}={x}_{i}^{k}+{v}_{i}^{k+1}$$

16 $${v}_{i}^{k+1}=\omega {v}_{i}^{k}+{c}_{1}{r}_{1,i}^{k}\left({p}_{i}^{k}-{x}_{i}^{k}\right)+{c}_{2}{r}_{2,i}^{k}\left({g}^{k}-{x}_{i}^{k}\right)$$

where ${x}_{i}^{k+1}$ and ${x}_{i}^{k}$ are the position vector of the ${i}^{th}$ solution in the ${\left(k+1\right)}^{th}$ and ${k}^{th}$ iteration; ${v}_{i}^{k+1}$ and ${v}_{i}^{k}$ are the velocity vector of the ${i}^{th}$ solution in the ${\left(k+1\right)}^{th}$ and ${k}^{th}$ iteration; ${p}_{i}^{k}$ represents the personal best position of the ${i}^{th}$ solution in the past $k$ iterations; ${g}^{k}$ represents the global best position of all solutions in the past $k$ iterations; $\omega$ is an inertia coefficient; ${c}_{1}$ and ${c}_{2}$ are individual learning factors and group learning factors. ${r}_{1,i}^{k}$ and ${r}_{2,i}^{k}$ are random values in the interval $\left[\text{0,1}\right]$ for the ${i}^{th}$ solution in the ${k}^{th}$ iteration.

### Two model evaluation indicators

In this study, the determination coefficient (R^2^) and Standard Deviation (SD) are used as the primary metrics to evaluate the goodness-of-fit and stability of these prediction models, respectively. R^2^ was chosen because it effectively quantifies the proportion of variance in pavement condition data explained by these probabilistic models, providing a clear measure of predictive accuracy. SD was selected to assess the stability of model predictions, as it directly reflects the dispersion of outputs. However, it is important to acknowledge the limitations of these metrics. R^2^ does not account for the magnitude of prediction errors and can be influenced by outliers or model complexity. Similarly, SD may not fully capture the distribution of uncertainties, especially in non-symmetric data. Alternative metrics, such as root mean squared error and confidence intervals, could provide additional insights and are recommended for future studies. Despite these limitations, R^2^ and SD remain appropriate for this research objectives, as they align with common practices in pavement condition modeling and provide a robust basis for comparing model performance.

Theoretical formula for R^2^^50^ is shown in Eq. (17).

17 $${\text{R}}^{2}=1-\frac{{\sum }_{n=1}^{N}{\left(\frac{1}{m}{\sum }_{m=1}^{M}{y}_{n,m}^{p}-{y}_{n}^{t}\right)}^{2}}{{\sum }_{n=1}^{N}{\left({y}^{a}-{y}_{n}^{t}\right)}^{2}}$$

where $N$ is the total number of predicted or true values; $n$ is the serial number of $N$; $M$ indicates the probabilistic prediction model is run $M$ times for a set of input values; $m$ is the serial number of $M$; ${y}_{n}^{t}$ denotes the ${n}^{th}$ true value;${y}^{a}$ is the average of all ${y}_{n}^{t}$ values; ${y}_{n,m}^{p}$ represents the ${n}^{th}$ predicted value in the ${m}^{th}$ run.

For a set of input values, the proposed probabilistic prediction model is run $M$ times to obtain $M$ output values, and then the standard deviation is computed. Finally, the mean of the $N$ standard deviations is computed as the prediction stability. The formula^50^ for prediction stability is shown in Eq. (18).

18 $$\text{SD}=\frac{1}{N}\sum_{n=1}^{N}\left\{\sqrt{\frac{1}{M-1}{\sum }_{m=1}^{M}{\left[{y}_{n,m}^{p}-\left(\frac{{\sum }_{m=1}^{M}{y}_{n,m}^{p}}{M}\right)\right]}^{2}}\right\}$$

## Results and discussion

### Selection of hyperparameters

For comparability, the hyperparameters of the BNN in the CS-BNN, BNN, GA-BNN and PSO-BNN models are the same. The hyperparameters include the number of hidden layers and neurons, optimizer, activation functions, learning rate, batch size, number of epochs, simulation times, dropout probability. Simulation times refer to the $M$ in Eqs. (17) and (18). The dropout probability is the probability that each neuron in the hidden layer is randomly dropped out during training and testing processes. One hidden layer is chosen for this study because it can model almost all nonlinear relationships. The hyperparameters in Table 3 were determined through iterative experimentation. A range of values for each hyperparameter was tested, and those maximizing model performance were selected. Specifically, the learning rate was evaluated in the range of [0.01, 0.2], and the dropout probability was tested in the range of [0.1, 0.5]. The final values (learning rate = 0.1, dropout probability = 0.3) were chosen based on their ability to maximize the R^2^.

**Table 3:** Hyperparameters of the BNN.

Hyperparameters	Selected values or items
Number of neurons in the hidden layer	12
Optimizer	Adam
Activation function	Leaky ReLU
Learning rate	0.1
Batch size	60
Number of epochs	10
Simulation times ($M$)	100
Dropout probability	0.3

For the selection of coefficient values in the CS, GA and PSO algorithms, several sets of alternative values were initially selected by referring to previous studies^36,44–46,51,52^, and then the final coefficient values were determined by several attempts based on the principle of maximizing the R^2^.

In the three algorithms, the number of solutions during the iterative search was set to 50 and the number of iterative searches was set to 50. Each element (weight or bias) in solutions has a value in the range of $[-2, 2]$, corresponding to the initial weights and biases obtained by random sampling from a truncated normal distribution.

In the CS algorithm, the probability (${P}_{a}$) of the random walk is 0.25. In the GA algorithm, the probabilities of crossover and mutation are 0.9 and 0.1, respectively. In the PSO algorithm, the inertia coefficient $\omega$ is 1; individual and group learning factors (${c}_{1}$, ${c}_{2}$) are both 1.5; and the range of velocity vector values (${v}_{i}^{k}$) is $[-0.2, 0.2]$.

### Numerical results

#### Results of the proposed prediction model (CS-BNN)

**Fig. 8:**
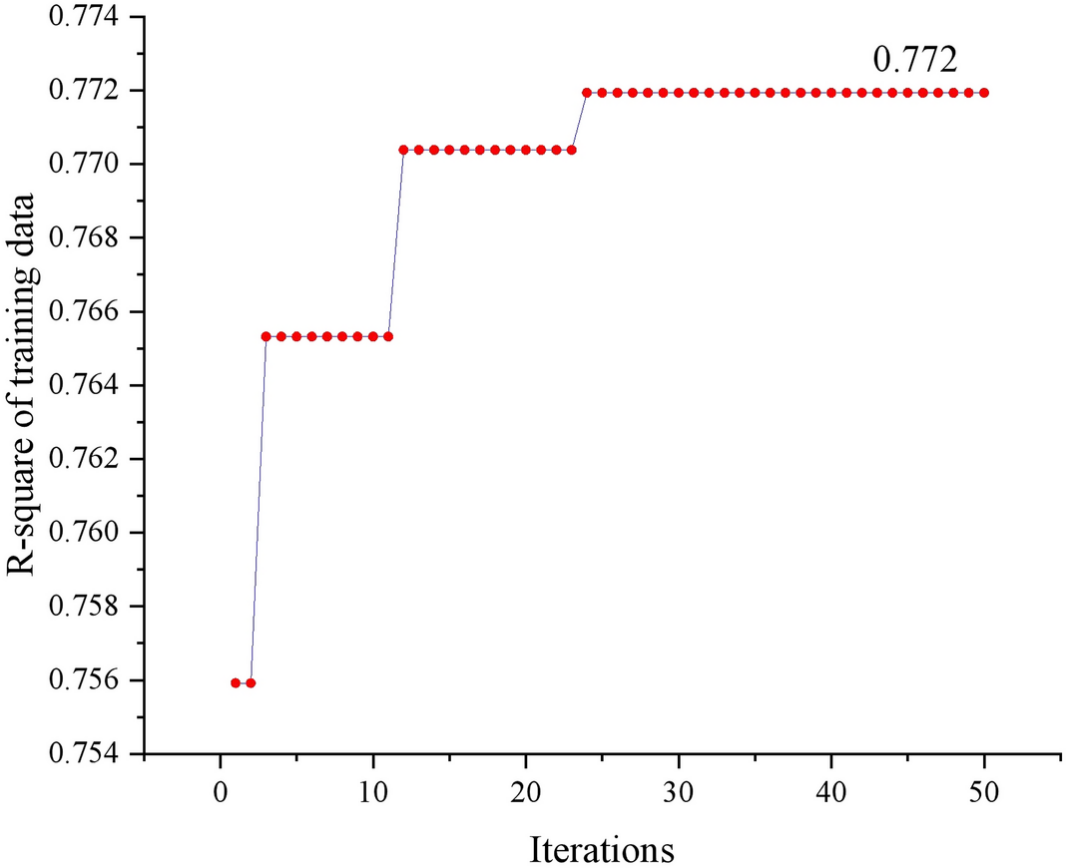
CS-BNN search processes based on training data.

With the objective of maximizing of the R^2^ obtained from the training data, the cuckoo algorithm performed 50 searches, and produced the results shown in Fig. 8. The maximum value of 0.772 is reached in the 24th search process, and the optimization result consistently remains at that value. This indicates that the number of iterative searches set to 50 is sufficient.

The corresponding searched initial weights and biases are shown in Table 4, totaling 145 values. The BNN model in this study has a total of 10 neurons in the input layer, 12 neurons in the hidden layer and 1 neuron in the output layer. Thus, the weights from the input layer to the hidden layer are 10*12 matrices and the biases are 12*1 matrices corresponding to the values of the 1st to 120th terms and the 121st to 132nd terms, respectively. The weights from the hidden layer to the output layer are 12*1 matrices and the bias is 1*1 matrix corresponding to the values of the 133rd to the 144th terms and the 145th term, respectively.

**Table 4:** Initial weights and biases searched from CS-BNN.

Layers	Parameter type	No.	Values
Input to hidden layer	Weights	1	− 0.565
		2	0.542
		…	…
		120	0.997
	Biases	121	1.656
		122	− 0.753
		…	…
		132	1.233
Hidden to output layer	Weights	133	0.591
		134	− 0.002
		…	…
		144	0.47
	Biases	145	0.284

**Fig. 9:**
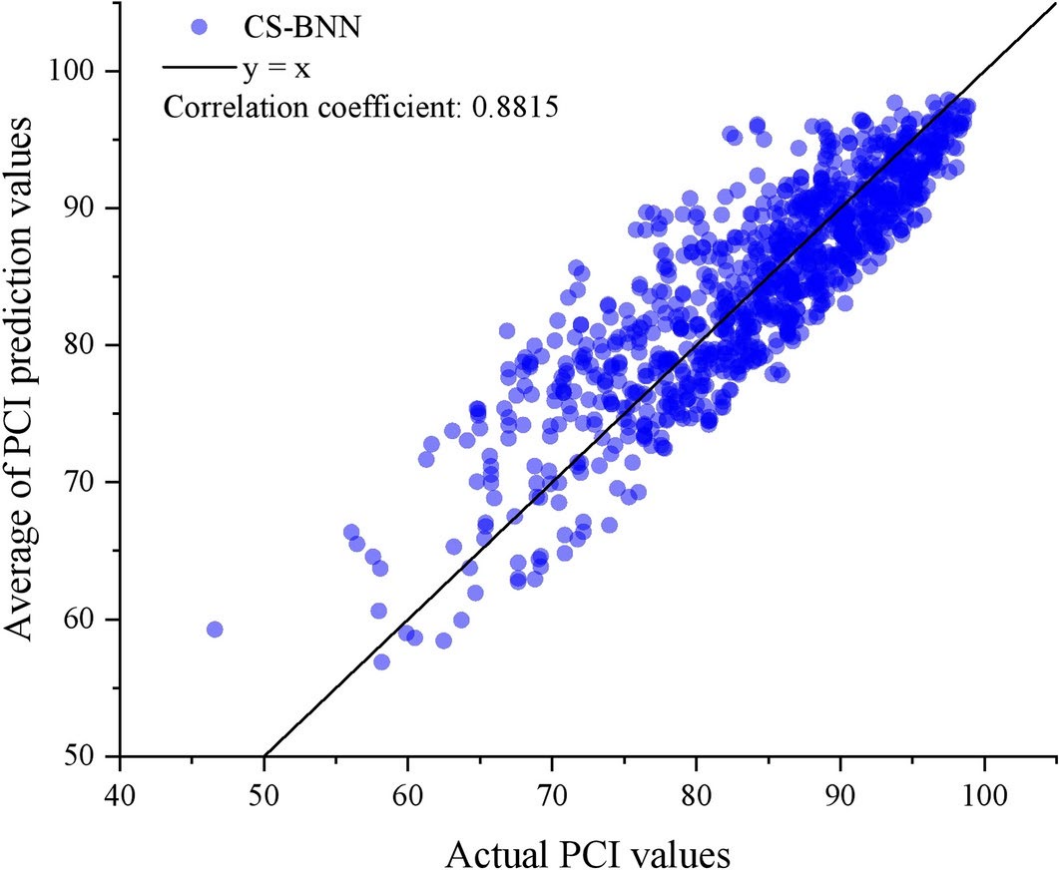
Comparison of the actual PCI values and the predicted mean values from the CS-BNN model.

Based on the searched initial weights and biases, the BNN model was retrained using the training data. Then, the R^2^ and stability of the retrained model were computed using the testing data. The R^2^ and stability (SD) values are 0.778 and 1.806, respectively. The relationship between the true PCI values and the predicted mean values of the testing data is shown in Fig. 9. Moreover, the correlation coefficient between the true PCI values and the predicted mean values is calculated to be 0.8815. Figure 9 shows the better correlation coefficient between the predicted and true PCI values of the CS-BNN model, demonstrating its potential to provide reliable predictions for pavement management decisions.

#### Superiority of the proposed prediction model

**Fig. 10:**
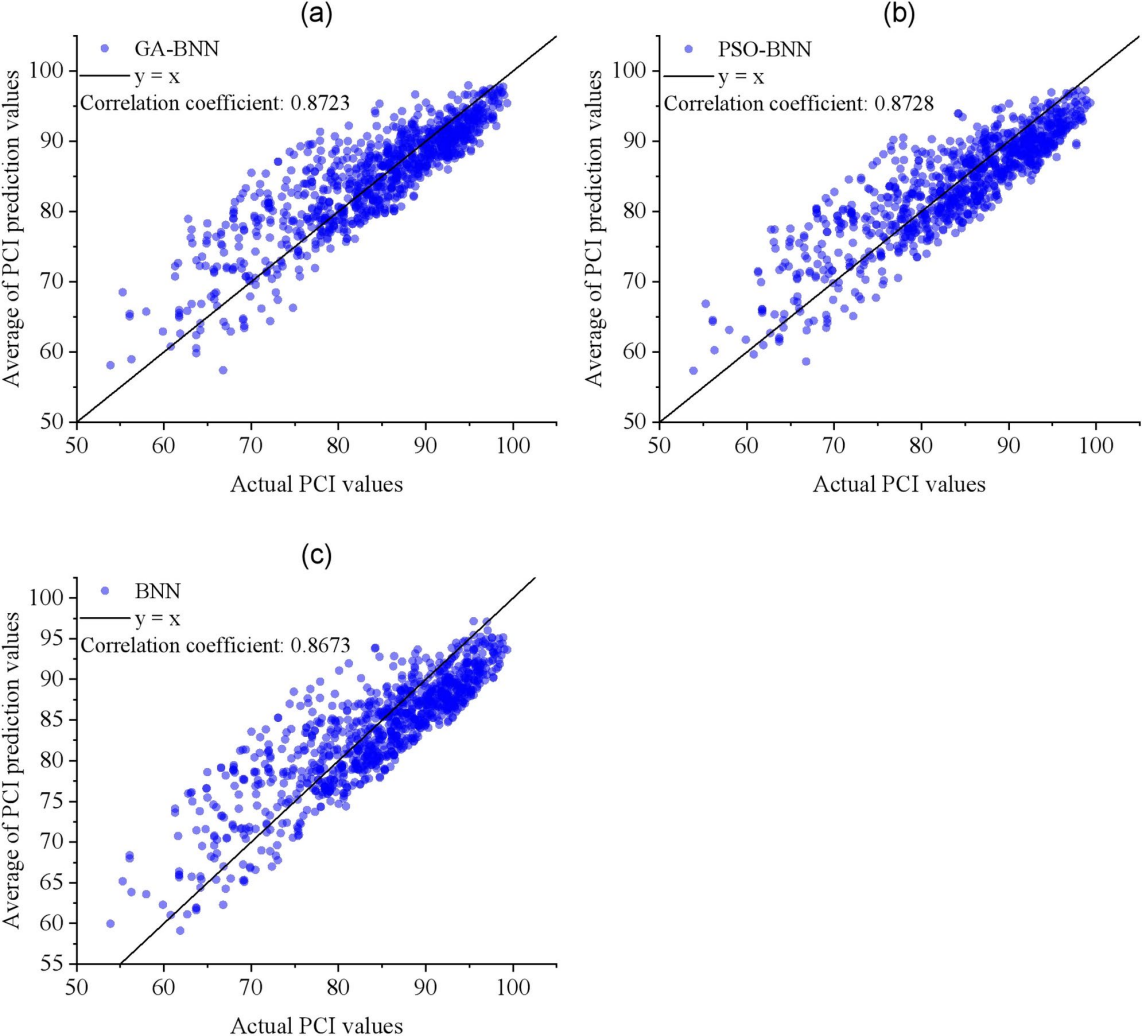
Comparison of the actual PCI values and the predicted mean values from (**a**) the GA-BNN model, (**b**) the PSO-BNN model, and (**c**) the BNN model.

**Fig. 11:**
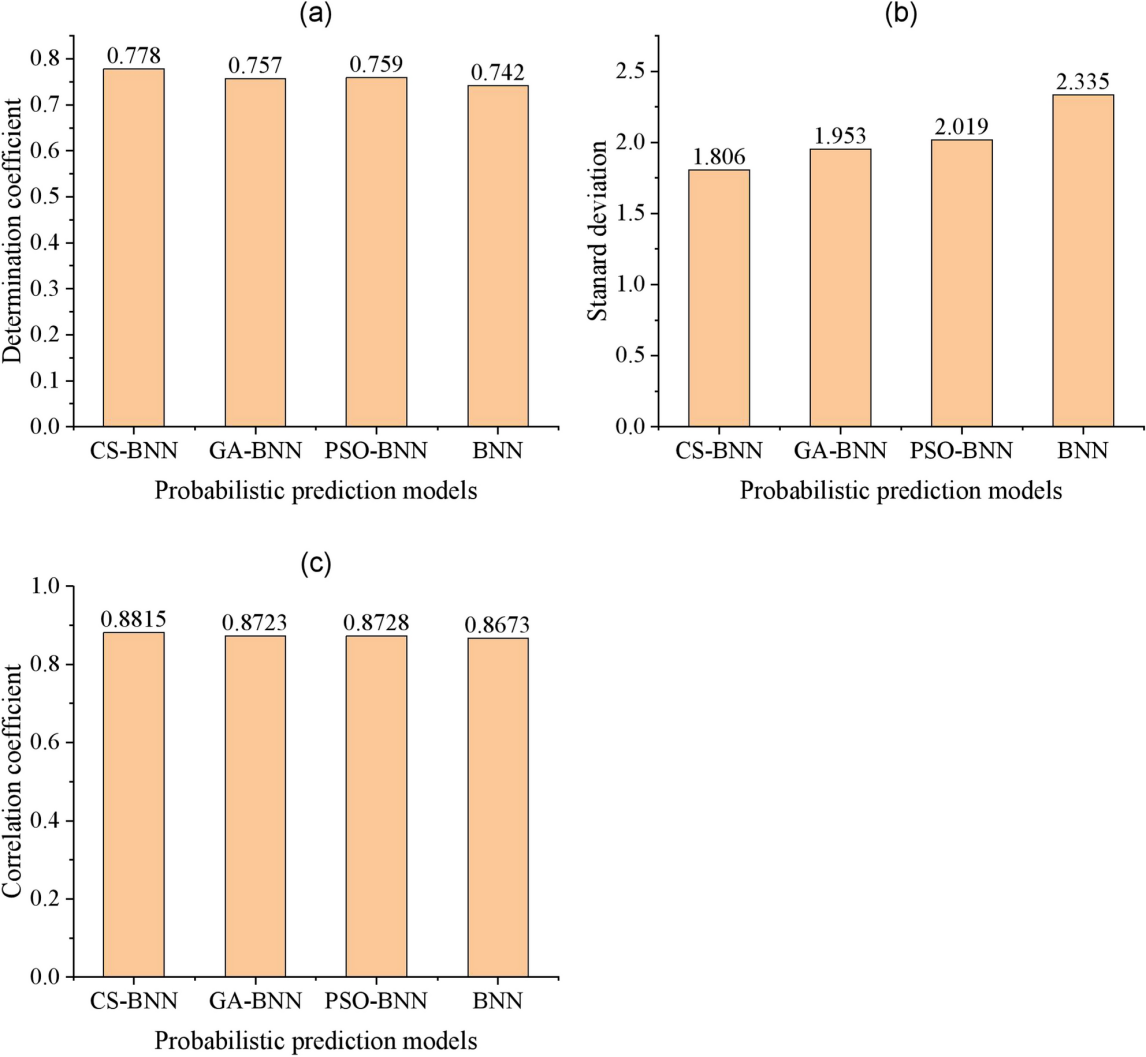
Comparison of four probabilistic prediction models in terms of (**a**) determination coefficient, (**b**) standard deviation, and (**c**) correlation coefficient.

In order to verify the superiority of the pavement deterioration prediction model based on CS-BNN, three comparison models were established. Using the same training data, three pavement deterioration prediction models based on BNN, GA-BNN and PSO-BNN were trained. Subsequently, the R^2^ and stability (SD) were computed using validation data. A larger R^2^ value means higher goodness-of-fit (predictive accuracy), while a smaller SD value indicates better stability. The relationship between the true PCI values and the predicted mean values from the GA-BNN, PSO-BNN, and BNN models is illustrated in Fig. 10. Correspondingly, the three correlation coefficients are calculated. The proposed CS-BNN model is compared with the three comparative models in terms of the R^2^, standard deviation, and correlation coefficient, as shown in Fig. 11. The larger R^2^ values and correlation coefficient values mean higher goodness-of-fit, while the smaller SD values indicate better stability.

According to Fig. 11, three conclusions can be obtained: (1) the goodness-of-fit and stability of CS-BNN, GA-BNN and PSO-BNN are better than that of the BNN model; (2) the goodness-of-fit and stability of the CS-BNN prediction model are better than the other three prediction models; and (3) as the goodness-of-fit decreases, the stability deteriorates roughly.

**Fig. 12:**
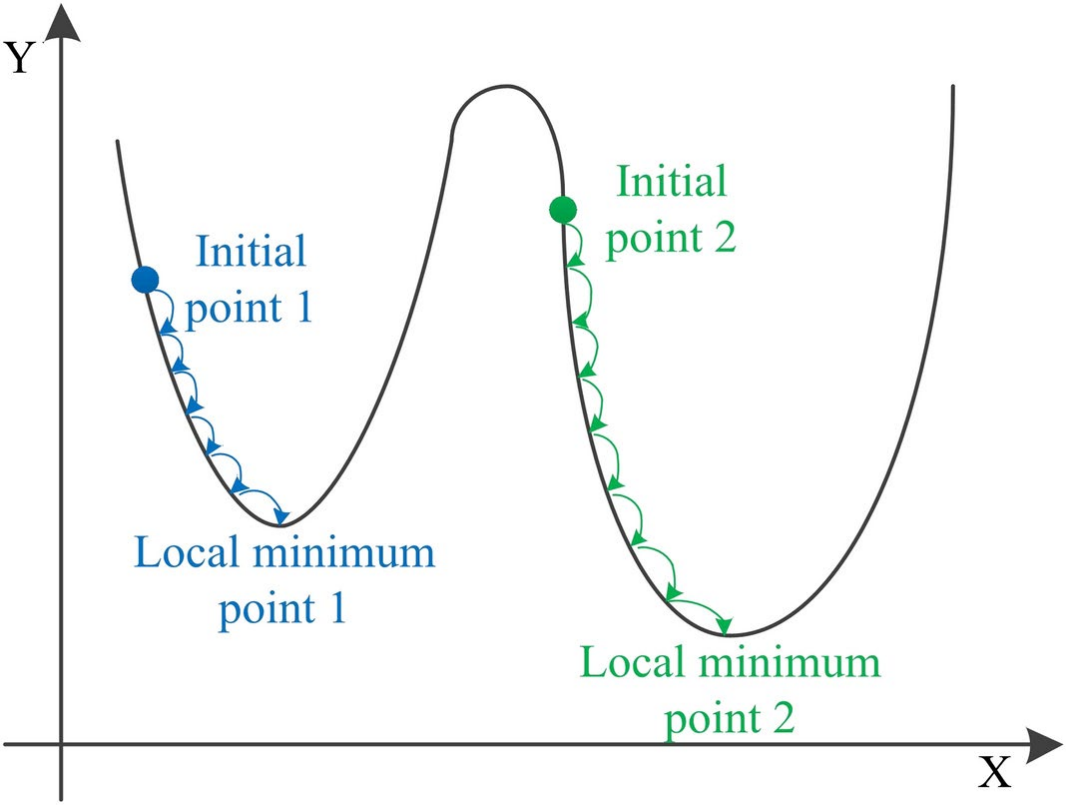
Relationship between the initial point and the searched minimum point.

For the first conclusion, the reason behind it was explored. In contrast to the BNN model, the CS-BNN, GA-BNN and PSO-BNN models have an extra step of searching for initial weights and biases. To better help readers understand the reason for the first conclusion, the search variable was assumed to be one-dimensional, as shown in Fig. 12. If the extra step is ignored, the randomly generated initial weights and biases are most likely to be the initial point 1. Then, the BNN model will converge to the local minimum point 1. Based on CS, GA, and PSO, the initial point 2 can be searched with high probability. So, the BNN model will converge to the local minimum point 2. This results in the superiority of the CS-BNN, GA-BNN and PSO-BNN over the BNN model.

For the second conclusion, two reasons behind it were explored. The first reason is that CS has fewer parameters than GA and PSO^45,46^. The second reason is the exploratory moves based on global search (Lévy flight) and local search (random walk), which are more efficient for large search spaces. It is these two reasons that cause CS-BNN to outperform GA-BNN and PSO-BNN prediction models. In addition, the second conclusion is able to demonstrate the superiority of the proposed prediction model.

**Fig. 13:**
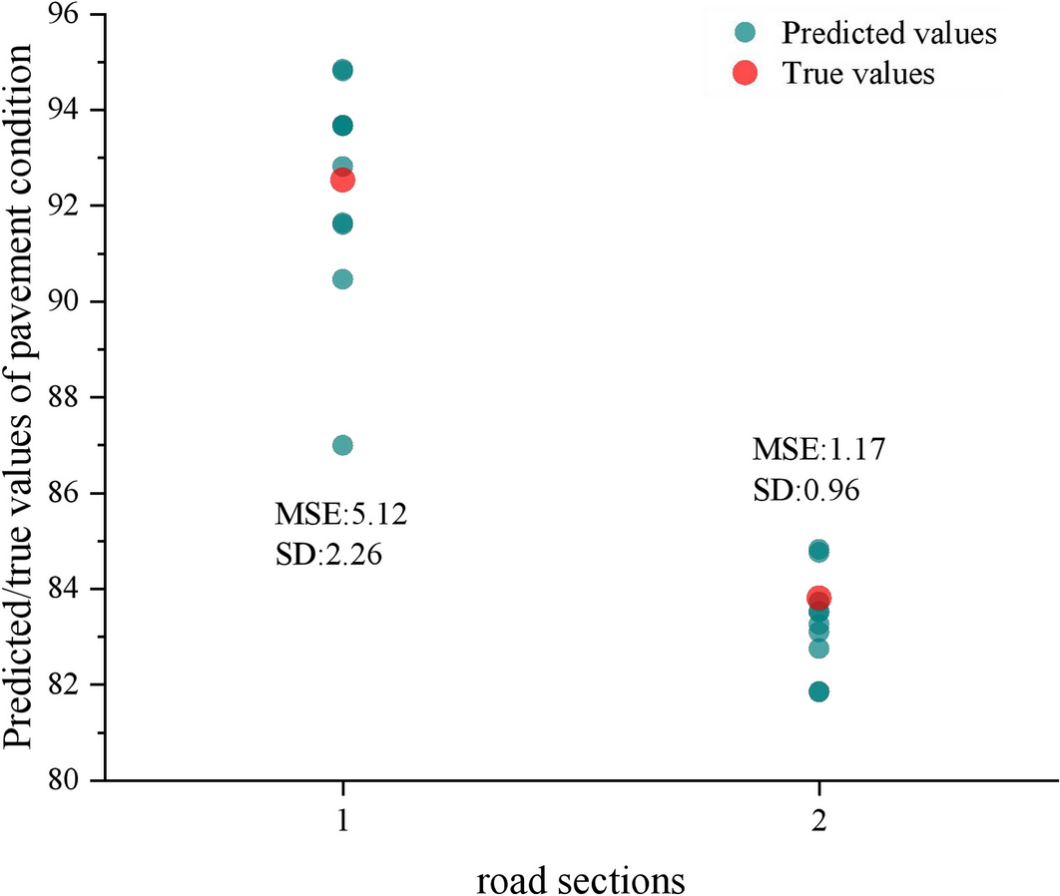
Relationship between predicted and true values for two road sections.

For the third conclusion, the mechanism behind it was also revealed. Data from two road sections were substituted into the trained CS-BNN model, and the computations were repeated 10 times. Then, the relationship between predicted and true values was plotted in Fig. 13. Due to the inability to calculate R^2^ for individual road sections, the Mean Square Error (MSE) was used to characterize the goodness-of-fit of individual road sections. The theoretical formula is shown in Eq. (19). The smaller the MSE value, the higher the goodness-of-fit. Comparing the relationship between the predicted and true values of road sections 1 and 2, it can be intuitively judged that when the goodness-of-fit is better, predicted values are closer to true values. In the meantime, the dispersion of predicted values decreases, and naturally the stability becomes better. On the contrary, the goodness-of-fit decreases, and the stability deteriorates roughly.

19 $$\text{MSE}=\frac{1}{N*M}{\sum }_{n=1}^{N}{\sum }_{m=1}^{M}{\left({y}_{n}^{t}-{y}_{n,m}^{p}\right)}^{2}$$

The superior goodness-of-fit and stability of the CS-BNN model have significant practical implications for pavement management. By providing more reliable predictions of pavement condition evolution, the model enables engineers to optimize maintenance timing by accurately identifying when interventions are needed, thereby preventing premature or delayed actions that could lead to higher costs or reduced pavement performance. Additionally, it allows for a better assessment of maintenance effectiveness by estimating the long-term impact of different strategies, supporting the selection of the most cost-effective solutions. Furthermore, the model improves budget planning by reducing uncertainty in cost estimations, enabling agencies to allocate resources more efficiently and prioritize high-impact projects. These benefits collectively demonstrate the potential of the CS-BNN model to enhance data-driven decision-making in pavement management.

#### Analysis of the proposed prediction model

As mentioned above, the CS algorithm has fewer parameters, i.e., one parameter—the probability (${P}_{a}$) of the random walk. In this study, ${P}_{a}$ had been taken to be 0.25. In order to analyze the effect of the ${P}_{a}$ on the proposed prediction model (CS-BNN), four additional had been selected: 0.05, 0.15, 0.35, and 0.45. Then, the goodness-of-fit and stability of the proposed prediction model under different ${P}_{a}$ values were computed separately, as shown in Table 5. It can be intuitively seen that the ${P}_{a}$ seems to have an insignificant effect on the goodness-of-fit and stability of the proposed model, which matches the findings of the previous study^45^.

**Table 5:** Evaluation metrics of the proposed model under different ${\text{P}}_{\text{a}}$ values.

${P}_{a}$	R^2^	SD
0.05	0.779	1.868
0.15	0.768	1.952
0.25	0.778	1.806
0.35	0.765	1.968
0.45	0.769	2.036

#### Sensitivity analysis of the CS-BNN model

**Fig. 14:**
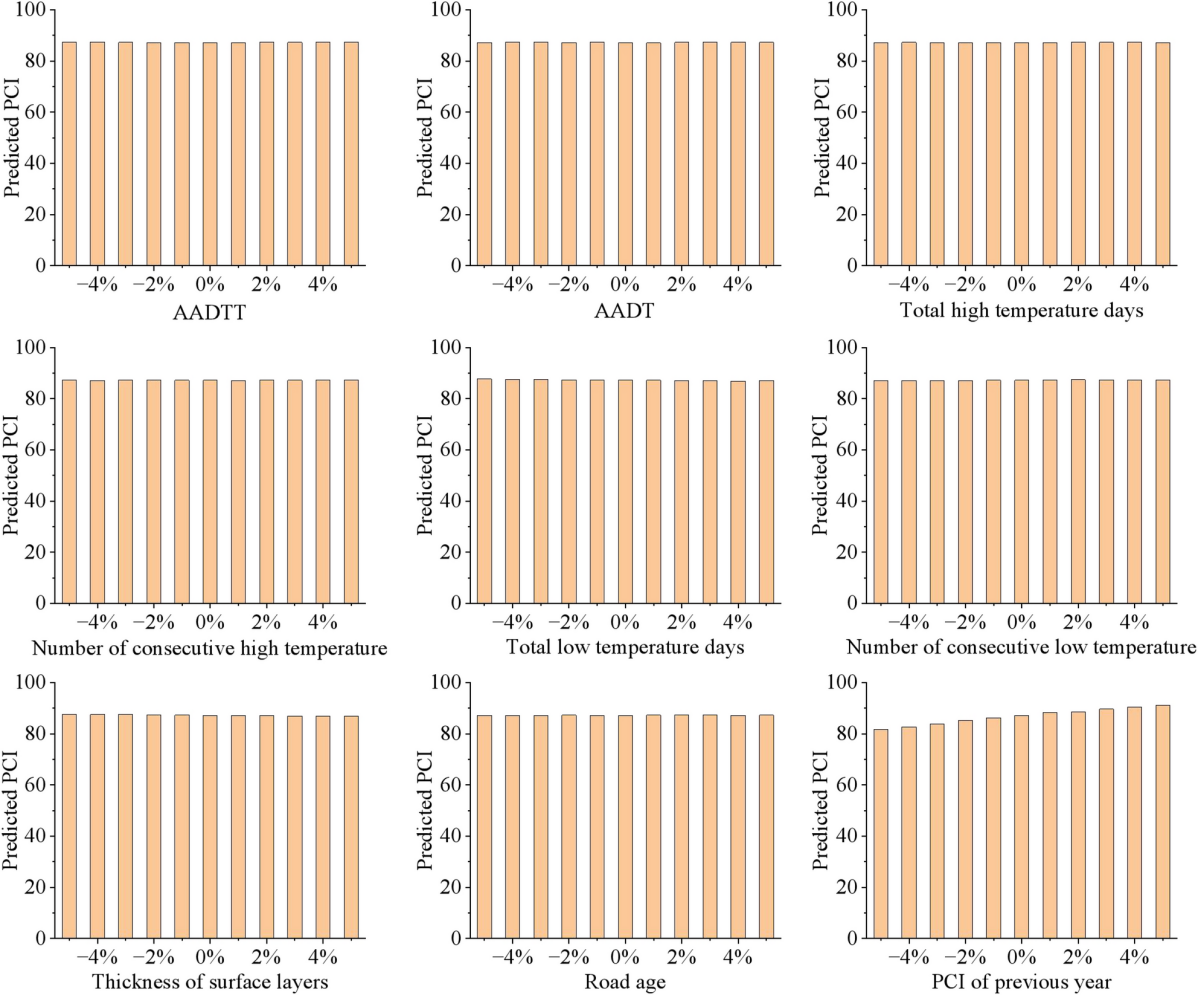
Sensitivity analysis of predicted PCI to ± 5% variations in input variables.

In order to verify the robustness of the CS-BNN model, small changes of -5% ~  + 5% were made to each numerical input variable, and the changes in the output values were observed, i.e., sensitivity analysis. In this case, the median of each input variable is used as the base case, and the predicted PCI values are shown in Fig. 14. The 0% in the horizontal coordinate indicates the selected median (base case) of input variables, and − 4%, − 2%, 2%, and 4% indicate a decrease of 4%, a decrease of 2%, an increase of 2%, an increase of 4%, respectively, on the basis of the base case. It can be seen that the predicted PCI values did not oscillate drastically when each of the 9 numerical input variables changed slightly, which can demonstrate the robustness of the CS-BNN model. In addition, the predicted PCI increase gradually as the PCI of previous year increases, suggesting that the PCI of previous year is relatively sensitive for the CS-BNN model compared to the other eight variables. The high sensitivity of the PCI of previous year can be attributed to its direct and strong relationship with the current pavement condition. Pavement performance is inherently time-dependent, and historical condition data provides a robust baseline for predicting future performance. In contrast, variables such as the total low temperature days have a more indirect impact, as their effects are often cumulative and long-term. The CS-BNN model prioritizes variables with a strong and direct relationship to the target variable, which explains why the PCI of previous year exhibits higher sensitivity. The high sensitivity of the PCI of previous year means that when collecting the PCI value, errors in that value should be minimized to improve the goodness-of-fit and stability of the CS-BNN prediction model.

## Conclusions and future work

This study proposed an improved probabilistic prediction model for pavement deterioration based on CS-BNN, which demonstrated superior performance in terms of goodness-of-fit and stability compared with GA-BNN, PSO-BNN and BNN models. The major findings in this study can be summarized as follows.

1. The pavement deterioration prediction model based on CS-BNN outperforms these based on GA-BNN, PSO-BNN and BNN in terms of the goodness-of-fit and stability.
2. The goodness-of-fit and stability of pavement deterioration probabilistic prediction models are roughly positively correlated.
3. The probability (${P}_{a}$) of random walk has an insignificant effect on the goodness-of-fit and stability of the proposed pavement deterioration prediction model.

The CS-BNN model’s superior performance in predicting pavement deterioration provides a more reliable tool for infrastructure managers. This improvement can lead to better decision-making in maintenance and rehabilitation scheduling, resource allocation, and long-term pavement management strategies. The positive correlation between the goodness-of-fit and the stability simplifies the model optimization process, as improving one metric simultaneously enhances the other, eliminating the need for trade-offs. The insensitivity to random walk probability simplifies the application of the CS-BNN model in practice. This reduces the complexity of model calibration and makes it more accessible for real-world implementation. The findings collectively contribute to advancing pavement management practices by providing a more accurate and robust tool for predicting pavement condition deterioration.

One of the key challenges in current pavement management practices is that existing prediction models often fail to account for uncertainties, leading to underestimation of maintenance costs and overestimation of post-maintenance performance. The proposed CS-BNN model addresses this issue by incorporating uncertainty quantification through its Bayesian framework, while simultaneously achieving superior goodness-of-fit and stability in predictions. This improvement enables more accurate estimation of required maintenance budgets and more realistic expectations of pavement performance after maintenance, ultimately supporting better decision-making for road engineers and infrastructure managers.

In future studies, it is worthwhile to extend the application of CS-BNN to other transportation infrastructures such as railroads, bridges and tunnels. Rail track degradation is influenced by factors such as dynamic loads, track geometry, and environmental conditions. The CS-BNN model could be adapted to predict rail track degradation, addressing challenges related to maintenance scheduling and safety. Bridge deterioration involves complex interactions between material aging, traffic loads, and environmental stressors. The CS-BNN model could be used to predict concrete cracking or steel corrosion, helping prioritize rehabilitation efforts and extend service life. Tunnel degradation often involves issues like lining cracks, water infiltration, and ground movement. The CS-BNN model could predict these defects, supporting proactive maintenance and reducing the risk of sudden failures.

Moreover, the CS algorithm can be coupled not only with BNN, but perhaps also with other deep learning algorithms to enhance the model performance, such as Long Short-Term Memory (LSTM), transformer, and so on. Pavement deterioration is a time-dependent process influenced by historical conditions (e.g., traffic loads, weather patterns). LSTM’s ability to capture temporal dependencies makes it ideal for modeling such sequential data, improving the accuracy of long-term predictions. Transformers excel at handling complex, non-linear relationships in data through their self-attention mechanisms. This capability is valuable for pavement deterioration prediction, where multiple factors (e.g., material properties, environmental conditions) must be considered simultaneously.

Due to insufficient years of modeling data, the current CS-BNN model can only predict PCI for the next year. To solve this problem, data from sufficient years should be collected as much as possible to develop a reliable multi-year PCI prediction model based on the CS-BNN theory in the future. Besides, there are 4 strategies to overcome the data limitations: synthetic data generation, transfer learning, data augmentation, collaborative data sharing. Techniques such as generative adversarial networks or Monte Carlo simulations can be used to generate synthetic pavement condition data, supplementing limited real-world data. Transfer learning can address data scarcity by leveraging pre-trained models from related domains (e.g., bridge or railroad deterioration) that have larger datasets. By fine-tuning these models on the available pavement data, the dependency on large amounts of pavement-specific data can be reduced while still achieving reliable predictions. Existing data can be augmented by introducing variations (e.g., noise, scaling) to simulate different conditions, increasing the diversity of the training dataset. Partnerships with other agencies or regions can facilitate the pooling of pavement condition data, expanding the dataset available.

## Supplementary Information

Supplementary Information.

## Data Availability

The data that support the findings of this study are available from the corresponding author upon reasonable request.
