# Supplementary material for: Enhanced probabilistic prediction of pavement deterioration using Bayesian neural networks and cuckoo search optimization
Source: Sci Rep. 2025 Mar 13;15:8665. doi: 10.1038/s41598-025-92469-9 (PMC11906751; doi:10.1038/s41598-025-92469-9)
Supplement: Supplementary file 1 — Supplementary Information. [file 41598_2025_92469_MOESM1_ESM.docx]

**Appendix A: Implementation Details of BNN, GA-BNN, and PSO-BNN models**

**A1. BNN model**

Except for the different sources of initial weights and biases, the BNN model here is the same as the BNN in the proposed CS-BNN model, with the structure shown in Figure 1.

Figure 1 The structure of BNN-based pavement deterioration prediction model

1. Initialization

The initial weights and biases are randomly sampled from a truncated normal distribution, which means that if the difference between a sampled value and the mean (i.e., 0) is greater than twice the standard deviation (i.e., 1), the sampled value is regenerated.

2. Monte Carlo Dropout

Dropout is applied during both training and testing with a probability of 0.3.

3. Hyperparameter settings

Optimizer: Adam

Activation function: Leaky ReLU

Batch size: 60

Number of epochs: 10

Simulation times for each input values during both training and testing: 100

**A2. Genetic Algorithm (GA)-BNN model**

Similar to the CS-BNN model, the GA is used to search the best initial weights and biases for BNN modeling. The BNN in the GA-BNN model is identical to the BNN model described above.

1. Initialization

The initial population of solutions (weights and biases) is randomly sampled from the truncated normal distribution.

2. Fitness function

The fitness of each solution is evaluated using the coefficient of determination (R^2^) of the BNN model trained with the corresponding weights and biases.

3. Crossover and mutation

Crossover: A single-point crossover is applied with a probability of 0.9.

Mutation: A solution is randomly selected with a probability of 0.1. Then, a gene locus is randomly selected for mutation on that solution.

The crossover and mutation operations are shown in Figure 6 of main text. To further facilitate readers’ understanding, the Python codes for crossover and mutation are as follows:

# Crossover operation

def Crossover(pop, pc):

[px, py] = pop.shape

newpop = np.zeros((px, py))

for i in range(0,px,2):

if np.random.rand()<pc: # pc=0.9

cpoint = round(np.random.rand()*py)

if cpoint == py:

cpoint = py-1

newpop[i,:] = np.concatenate((pop[i,:cpoint], pop[i+1,cpoint:]))

newpop[i+1,:] = np.concatenate((pop[i+1,:cpoint], pop[i, cpoint:]))

else:

newpop[i,:] = pop[i,:]

newpop[i+1,:] = pop[i+1,:]

return newpop

# Mutation operation

def Mutation(pop, pm):

[px, py] = pop.shape

for i in range(0, px):

if np.random.rand()<pm: # pm=0.1

mpoint = round(np.random.rand()*py)

if mpoint == py:

mpoint = py-1

pop[i, mpoint] = random.uniform(Xmin,Xmax)

return pop

4. Population regeneration (elite selection)

For the new population obtain through crossover and mutation, its fitness values are compared with that of the previous generation one by one, and the solution with better fitness is kept. To further facilitate readers’ understanding, the Python codes for elite selection are as follows:

def func_bestNestPop(nestPop, fitness, new_nestPop, fitness_New):

index = np.where(fitness>fitness_New)

nestPop[index,:] = new_nestPop[index,:]

fitness[index] = fitness_New[index]

return nestPop, fitness

5. Hyperparameter settings

Population size: 50

Maximum generations: 50

**A3. Particle Swarm Optimization (PSO)-BNN model**

Similar to the CS-BNN model, the PSO is used to search the best initial weights and biases for BNN modeling.

The BNN in the PSO-BNN model is identical to the BNN model described above. The initialization, fitness function of the PSO-BNN model are the same as the GA-BNN model. The velocity vector and position vector update of the PSO has been explained in Equations (15) and (16) as well as Figure 7 in the main text, so they will not be repeated.

Hyperparameter settings

Population size: 50

Maximum generations: 50

Inertia coefficient: 1

Individual learning factor: 1.5

Group learning factor: 1.5
